# Effective Suppression of Pathological Synchronization in Cortical Networks by Highly Heterogeneous Distribution of Inhibitory Connections

**DOI:** 10.3389/fncom.2016.00109

**Published:** 2016-10-18

**Authors:** Hisashi Kada, Jun-Nosuke Teramae, Isao T. Tokuda

**Affiliations:** ^1^Department of Mechanical Engineering, Ritsumeikan UniversityKusatsu-Shi, Japan; ^2^Department of Bioinformatic Engineering, Graduate School of Information Science and Technology, Osaka UniversitySuita, Japan

**Keywords:** spontaneous firing, cortical network, lognormal distribution, excitatory and inhibitory connections, heterogeneity, synchronization

## Abstract

Even without external random input, cortical networks *in vivo* sustain asynchronous irregular firing with low firing rate. In addition to detailed balance between excitatory and inhibitory activities, recent theoretical studies have revealed that another feature commonly observed in cortical networks, i.e., long-tailed distribution of excitatory synapses implying coexistence of many weak and a few extremely strong excitatory synapses, plays an essential role in realizing the self-sustained activity in recurrent networks of biologically plausible spiking neurons. The previous studies, however, have not considered highly non-random features of the synaptic connectivity, namely, bidirectional connections between cortical neurons are more common than expected by chance and strengths of synapses are positively correlated between pre- and postsynaptic neurons. The positive correlation of synaptic connections may destabilize asynchronous activity of networks with the long-tailed synaptic distribution and induce pathological synchronized firing among neurons. It remains unclear how the cortical network avoids such pathological synchronization. Here, we demonstrate that introduction of the correlated connections indeed gives rise to synchronized firings in a cortical network model with the long-tailed distribution. By using a simplified feed-forward network model of spiking neurons, we clarify the underlying mechanism of the synchronization. We then show that the synchronization can be efficiently suppressed by highly heterogeneous distribution, typically a lognormal distribution, of inhibitory-to-excitatory connection strengths in a recurrent network model of cortical neurons.

## 1. Introduction

Sustained asynchronous irregular activity of cortical neurons is commonly observed in cell cultures (Gross et al., 1982; Plenz and Aertsen, 1996; Marom and Shahaf, 2002), *in vitro* (Mao et al., 2001; Shu et al., 2003b), and *in vivo* (Timofeev et al., 2000) even in the absence of external stimuli. The spontaneous asynchronous activity is, as a ground state of the cortex, assumed to be involved in various significant computations realized in cortex, including sensory perception (Arieli et al., 1996; Tsodyks et al., 1999), working memory (Fuster, 1995; Wang, 2002; Compte, 2006), signal processing (Kenet et al., 2003), and transmissions (Destexhe and Contreras, 2006; Kumar et al., 2010). While the underlying mechanism for the cortical network to generate and maintain the spontaneous asynchronous state has not been fully understood yet, theoretical studies of random networks with balanced excitatory and inhibitory activities showed that random networks of leaky integrate-and-fire neurons without external inputs can realize the asynchronous irregular state under a certain condition (Vogels and Abbott, 2005; Kumar et al., 2008).

Considering the latest findings of heterogeneous features of synaptic strengths of cortical neurons (Song et al., 2005; Lefort et al., 2009; Avermann et al., 2012; Buzsáki and Mizuseki, 2014), recent numerical and theoretical studies revealed that the asynchronous irregular state is robustly and spontaneously realized in networks of biologically plausible leaky integrate-and-fire neurons with largely relaxed condition from the previous ones (Teramae et al., 2012; Ikegaya et al., 2013; Kriener et al., 2014). Moreover, asynchronous activities of neurons realized in these studies faithfully share various properties with the sustained activity actually observed *in vivo*, such as high-irregularity (Softky and Koch, 1993; Stiefel et al., 2013), extremely low firing rate (Hromádka et al., 2008; Mizuseki and Buzsáki, 2013), high-conductance membrane potential with large fluctuation (Wilson and Kawaguchi, 1996; Destexhe et al., 2001), and persistent UP state of membrane potential (Steriade et al., 2001; Destexhe et al., 2003; Shu et al., 2003a). In these models, amplitudes of excitatory postsynaptic potentials (EPSP) for excitatory-to-excitatory connections follow a long-tailed distribution, such as the lognormal distribution, with many weak and a few extremely strong synapses, which allows networks to generate and stably maintain the asynchronous irregular activity (Teramae et al., 2012).

Another significant feature of synaptic connectivity of local cortical circuit (Song et al., 2005), however, has been rarely considered in the previous studies of spontaneous asynchronous state. Namely, the synaptic connectivity in local cortical circuit is highly non-random. Bidirectional connections between cortical neurons are reported to exist more commonly than expected by chance in a random network and strengths of synapses are positively correlated between pre- and post-synaptic neurons, implying that strong synapses are more clustered than the majority of weak synapses. The clustered connectivity between strong synapses may excessively enhance mutual excitation among neurons in these clusters, which may force the spontaneous activity to increase its firing rate and as a consequence induce pathological synchronization among spikes. It remains unclear how the cortical network suppresses the pathologically strong synchronization induced by the clustered synaptic connectivity with the long-tailed EPSP distribution.

In this study, we propose a plausible mechanism for suppressing the pathological synchronization in cortical networks with clustered synaptic connectivity with the long-tailed EPSP distribution. First, we reveal that the synchronization is indirectly induced by common synaptic inputs from inhibitory-to-excitatory connections rather than directly induced by the excessive mutual excitation among the clustered neurons. Next, we show that highly heterogeneous distribution of synaptic strengths for inhibitory-to-excitatory connections, which is similar to the heterogeneous connectivity experimentally observed for excitatory-to-excitatory connections (Song et al., 2005; Lefort et al., 2009; Avermann et al., 2012; Buzsáki and Mizuseki, 2014), efficiently suppresses the pathological synchronization. We also numerically confirm that a moderate amount of heterogeneity given by *Gaussian* distribution of the inhibitory synaptic strengths is not sufficient to suppress the synchronization. Moreover, we show that heterogeneity of the synaptic strengths in neither excitatory-to-inhibitory nor inhibitory-to-inhibitory connections effectively suppresses the synchronization. This result suggests a novel role of the heterogeneous inhibitory-to-excitatory connections with extremely strong inhibition (Miles and Wong, 1984; Chapeton et al., 2012) in realization of robust cortical state and its computation.

The rest of the paper is organized as follows. In Section 2, we shortly describe the neuron and network models with explanation of analysis used in the paper. In Section 3.1, by introducing a finite correlation among EPSPs between bi-directionally connected excitatory neurons, we demonstrate that the experimentally observed synaptic connectivity, i.e., long-tailed EPSP distribution with the positive correlation, actually induce pathologic synchronization in the spontaneously sustained activity. In order to clarify the origin of the synchronization, in Section 3.2, we study a simplified feed-forward network model, in which target neurons receive common synaptic inputs from neurons in the previous layer. Section 3.3 provides a possible mechanism to suppress pathological synchronization and recover stable asynchronous irregular state by introducing a highly heterogeneous distribution to inhibitory connections. Finally, in Section 4, we summarize our results and discuss possible relationship of our findings to cortical organization.

## 2. Methods

### 2.1. Single neuron model

The dynamics of individual neuron is described by a conductance-based leaky integrate-and-fire model:

(1) $$\begin{array}{l} \frac{d\upsilon }{dt}=-\frac{1}{\tau_{m}}\left(\upsilon -V_{L}\right)-g_{E}\left(\upsilon -V_{E}\right)-g_{I}\left(\upsilon -V_{I}\right), \end{array}$$

where υ represents membrane potential, τ_*m*_ is membrane time constant, and *V*_*L*_, *V*_*E*_, and *V*_*I*_ are reversal potential of leak, excitatory and inhibitory postsynaptic currents, respectively. We use τ_*m*_ = 20 ms for excitatory neurons, τ_*m*_ = 10 ms for inhibitory neurons, *V*_*L*_ = −70, *V*_*E*_ = 0, and *V*_*I*_ = −80 mV. The excitatory and inhibitory synaptic conductances normalized by the membrane capacitance, *g*_*E*_ and *g*_*I*_, evolve with the following equation:

(2) $$\frac{dg_{X}}{dt}=-\frac{g_{X}}{\tau_{s}}+\sum_{j}G_{X,j}\sum_{s_{j}}\delta (t-s_{j}-d_{j}),\;X=E,I,$$

where the indices *X* = *E* and *X* = *I* are for excitatory and inhibitory conductances, respectively. δ(*t*) represents the delta function, *G*_*X, j*_, *d*_*j*_, and *s*_*j*_ are synaptic weight, delay, and spike timing of synaptic input from the *j*-th neuron, respectively. The decay time constant τ_*s*_ is set to 2 ms for both excitatory and inhibitory conductances. The synaptic delays *d*_*j*_ are chosen randomly from a uniform distribution between *d*_0_−1 and *d*_0_+1 ms, where the mean value is set as *d*_0_ = 2 ms for excitatory-to-excitatory connections and *d*_0_ = 1 ms for the others. The spike threshold is *V*_*thr*_ = −50 mV and υ is reset to *V*_*r*_ = −70 mV after the spiking. The refractory period is 1 ms. These parameters are based on the ones of Teramae et al. (2012). The Euler's method is used to integrate the differential Equations (1, 2) with a time step of 0.01 ms.

### 2.2. Organization of recurrent network model of cortex

The network model consists of *N*_*E*_ = 10, 000 excitatory neurons and *N*_*I*_ = 2000 inhibitory neurons. For pairs of excitatory neurons, probabilities of unidirectional connections and bidirectional connections are *P*_*uni*_ = 0.123 and *P*_*bi*_ = 0.0542, respectively, based on the physiological measurements (Song et al., 2005). Synaptic weights *G*_*E, j*_ (*j*∈*E*) for unidirectional connections are distributed such that the amplitudes of EPSPs *x* measured from the resting membrane potential obey a lognormal distribution,

(3) $$\begin{array}{l} p\left(x\right)=\frac{\exp\left[-{\left(\log x-\mu \right)}^{2}/2\sigma_{N}^{2}\right]}{\sqrt{2\pi }\sigma_{N}x}, \end{array}$$

where μ and σ_*N*_ represent mean and standard deviation of the variable's natural logarithm. We use values $\mu -\sigma_{N}^{2}=\log\left(0.2\right)$ and $\sigma_{N}^{2}=1.0$ to replicate experimentally observed long-tailed distributions of the amplitudes of EPSPs (Song et al., 2005). Any unrealistic value of *G*_*E, j*_ that gives amplitude of EPSP larger than 20 mV is discarded and we select a new value from the distribution.

In order to introduce the positive correlation of synaptic strengths measured between a pair of bidirectionally connected excitatory neurons, synaptic weights *G*_*j, k*_ and *G*_*k, j*_ (*j, k*∈*E*: bidirectionally connected excitatory neurons) are chosen such that amplitudes of the pair of EPSPs obey correlated random variables described as:

(4) $$\begin{array}{r} \begin{array}{c} x_{1}=\exp\left[\mu +\sigma_{N}\left(\sqrt{1-a}y_{1}+\sqrt{a}y_{3}\right)\right],\\ x_{2}=\exp\left[\mu +\sigma_{N}\left(\sqrt{1-a}y_{2}+\sqrt{a}y_{3}\right)\right], \end{array} \end{array}$$

where *y*_1_, *y*_2_, and *y*_3_ are independent *Gaussian* variables with zero mean and unit variance. Each of *x*_1_ and *x*_2_ obeys the lognormal distribution of Equation (2), where their correlation is derived analytically as $R=\left\{e^{a\sigma_{N}^{2}}-1\right\}/\left\{e^{\sigma_{N}^{2}}-1\right\}$. By changing the parameter *a*, the correlation *R* can be controlled. Physiologically measured value of the correlation is, for instance, *R* = 0.36 for rat visual cortex (Song et al., 2005).

Based on the previous study (Teramae et al., 2012), constant values of *G*_*E, j*∈*I*_ = 0.018, *G*_*I, j*∈*E*_ = 0.002, and *G*_*I, j*∈*I*_ = 0.0025 are used for excitatory-to-inhibitory, inhibitory-to-excitatory, and inhibitory-to-inhibitory connections, respectively. The connection probabilities of excitatory-to-inhibitory, inhibitory-to-excitatory and inhibitory-to-inhibitory neurons are *P*_*EI*_ = *P*_*bi*_+*P*_*uni*_/2 = 0.1157, *P*_*IE*_ = *P*_*II*_ = (*N*_*E*_/*N*_*I*_)*P*_*EI*_ = 0.5785, respectively. Excitatory-to-excitatory synaptic transmissions fail with a rate *p*_*E*_ = *b*/(*b*+*EPSP*), where *b* = 0.1 mV (Lefort et al., 2009). In order to initiate spontaneously maintained ongoing state, we apply transient external *Poisson* spike trains with 1 Hz to all neurons only during the initial duration of 100 ms. The total simulation time including the initial transient is set to 2100 ms. From time interval between 500 and 2100 ms, dynamic quantities, e.g., firing frequencies, index of synchronized firing, strength of common inhibitory input as explained in the followings, are computed.

### 2.3. Feed-forward network model

A feed-forward network model consisting of two layers of neurons is constructed to study the underlying mechanisms of correlation-induced synchrony. The input layer consists of 10, 000 excitatory and 2000 inhibitory neurons, whereas the output layer consists only of 1000 excitatory neurons. The connection probabilities from excitatory and inhibitory neurons in the input layer to neurons in the output layer are *P*_*EE*_ = 0.1157 and *P*_*IE*_ = 0.5785, respectively. The connection weights are set to be the same with those of the recurrent network model explained in the previous subsection.

Inhibitory neurons in the input layer generate mutually correlated *Poisson* spike trains as follows. First, we generate a single *Poisson* process with a constant firing rate and make each spike of the process be shared by randomly chosen 1% of the inhibitory neurons. The random selections are different from one another. Then, in addition to the randomly shared spikes, each neuron generates independent *Poisson* spikes so that its firing rate becomes 30 Hz in average. Frequency of the shared spikes ranges from 0 to 25 Hz. Excitatory neurons in the input layer generate independent *Poisson* spike trains, where their firing rate is adjusted so that excitatory neurons in the output layer fire with an average rate of 3 Hz.

### 2.4. Index of synchronized firing

We quantify the level of synchronization between spikes based on the cross-correlogram (CCG) of randomly chosen 1000 neurons. The CCG is calculated as a histogram of spike-time intervals (bin size: 1 ms, time lag: ±20 ms) for all pairs of the 1000 neurons. Since existence of a peak in the CCG indicates synchronized firings, we define the synchronization index as a normalized height of the peak as

(5) $$\begin{array}{l} SI=\frac{M-A}{M}, \end{array}$$

where *M* is the maximum and *A* is the average of the CCG.

### 2.5. Strength of common inhibitory input

As an index to measure the cause of synchrony, the strength of the common inhibitory input to excitatory neurons is quantified. The common inhibitory input is derived by time series of the inhibitory synaptic conductances *g*_*I*_ averaged over excitatory neurons as {ḡ_*I*_(*t*_*i*_):*i* = 1, 2, …, *T*}. Then, as the quantity to measure the signal strength, standard deviation of the averaged signal is computed with respect to the sampling time points as

(6) $$CI=\sqrt{\frac{1}{T}\sum_{i}^{T}\{{\bar{g}}_{I}(t_{i})-\langle {\bar{g}}_{I}\rangle {\}}^{2}},$$

where $\langle {ḡ}_{I}\rangle =\frac{1}{T}\overset{T}{\underset{i}{\sum }}{ḡ}_{I}\left(t_{i}\right)$ stands for time-average of the common inhibitory input.

## 3. Results

### 3.1. Correlation-induced pathological synchronization in the recurrent network model of cortex

**Figure 1:**
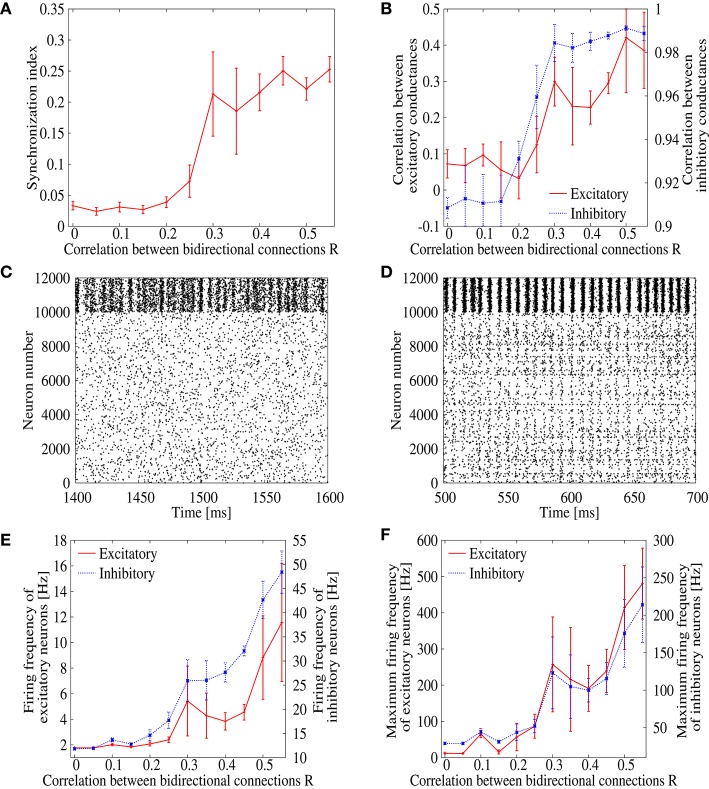
**(A)** Dependence of the synchronization index on correlation parameter *R*, which measures correlation of EPSPs between bidirectionl connections. The error bars indicate standard deviation of 5 realizations started from different random initial conditions. **(B)** Dependence of the correlation between inhibitory (blue) and excitatory (red) synaptic conductances of excitatory neurons on the correlation parameter *R*. **(C,D)** Raster plot representing the firing pattern of *R* = 0 **(C)** and *R* = 0.35 **(D)**. For excitatory neurons, spikes are indicated from neuron number 0 to 9999, whereas they are indicated from neuron number 10, 000 to 11, 999 for inhibitory neurons. **(E)** Dependence of the mean firing frequencies of excitatory (red) and inhibitory (blue) neurons on the correlation parameter *R*. **(F)** Dependence of the maximum firing frequencies of excitatory (red) and inhibitory (blue) neurons on the correlation parameter *R*.

We first evaluate synchronized firing of spontaneously sustained state in the recurrent network model of cortical circuits. We prepare networks with various values of correlation *R* between EPSP amplitudes of bi-directionally connected pair of excitatory neurons. We then numerically solve Equations (1) and (2) on these networks to obtain the ongoing firing state and measure their synchronization indices (Figure 1A). In networks with low bidirectional correlation, *R* < 0.25, the synchronization index is low, *SI* ≃ 0.05, where asynchronous ongoing activity is realized in a stable manner. Firing rate of the ongoing state is also kept low in this region (Figures 1E,F). The synchronization index, however, rapidly increases as the correlation *R* increases from *R* ≃ 0.25. In the network with *R* = 0.35, i.e., the value close to experimentally observed one for rat visual cortex, the synchronization index reaches *SI* = 0.19, which is significantly higher than the one, *SI* ≃ 0.05, observed with low correlation. Figures 1C,D shows the raster plot of the ongoing state in the networks with *R* = 0.0 and 0.35. The spike timings are highly correlated among neurons in the network with *R* = 0.35, whereas uncorrelated neural firings are observed with *R* = 0.0. This implies that normal firing state of the cortex is replaced by pathological synchronization among neurons as bidirectional correlation is introduced to the model networks, even though the bidirectional connections are considered biologically plausible. It should be noted that the inhibitory neurons are more clearly synchronized with each other compared to the excitatory neurons even with *R* = 0.0. This is however consistent with the experimental observation (Hasenstaub et al., 2005). Another note is that the large error-bar of Figure 1A at *R* = 0.25 indicates coexistence of synchronization/desynchronization states around there.

**Figure 2:**
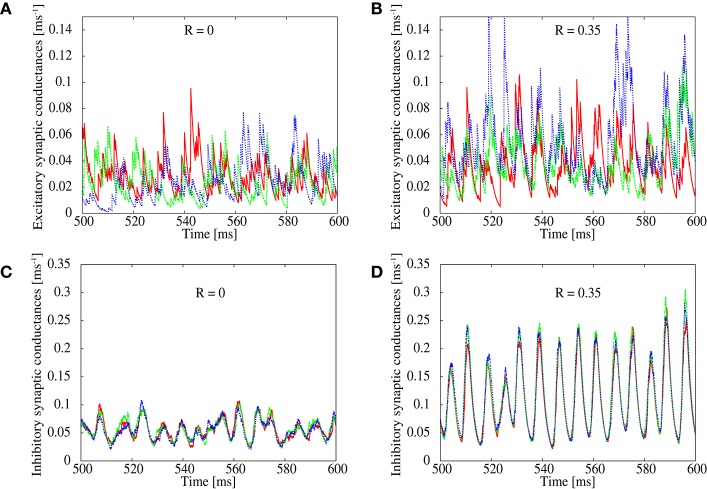
**Simultaneous plots of excitatory (top) and inhibitory (bottom) conductances of excitatory neurons**. The correlation parameter is set to *R* = 0 in **(A,C)** and *R* = 0.35 in **(B,D)**.

In order to study details of the pathological synchronization, we measure temporal development of excitatory and inhibitory conductances on several excitatory neurons during the ongoing state in the networks with *R* = 0.0 and 0.35 (Figure 2). We can see that inhibitory conductances are large and highly correlated with each other on different excitatory neurons especially when *R* = 0.35 (Figure 2D), whereas excitatory conductances on the same neurons are weak and relatively distributed when *R* = 0.0 (Figure 2C). This finding is also supported by Figure 1B which plots correlation between the inhibitory conductances as a function of *R*. For *R* > 0.25, relatively high correlation is indicated among inhibitory conductances on different excitatory neurons. It has been reported in *in vivo* study (Hasenstaub et al., 2005) that inhibitory conductances tend to be synchronized with each other. The correlation, however, may not exceed 0.5 as shown in Figure 1B.

The present result therefore implies that strongly correlated inhibitory inputs, rather than excitatory inputs, drive neurons toward the pathological synchronization. It has been well studied that a common drive can easily induce strong synchronization among nonlinear systems including neurons (Mainen and Sejnowski, 1995; Teramae and Tanaka, 2004; Galán et al., 2006; de La Rocha et al., 2007; Doiron et al., 2016). In our network, the common inhibitory drive is kept weak as far as the correlation parameter *R* is small, because firing rate of inhibitory neurons is kept low for a small *R* (Figures 1E,F). As *R* is increased, however, due to an increase in inhibitory firing rate as an outcome of excessive recurrent excitation mediated by the bidirectional excitatory connections, strength of the common inhibitory drive is increased and as a consequence induces strong pathological synchronization to the population of neurons in the network. The present finding agrees with other studies reporting that increase in firing rates leads to an increase in synchrony in cortical networks (Brunel and Hakim, 1999; Brunel, 2000; Mazzoni et al., 2008). Although, the network statistics are set to be closer to the real ones, the observed synchronized dynamics is significantly different from the asynchronous ongoing state observed in normal cortex.

### 3.2. Suppression of pathological synchronization in a simplified feed-forward network model

Results of the previous subsection suggest a possibility that the pathological synchronization can be efficiently suppressed by reduction of strong correlation among inhibitory inputs on excitatory neurons. In order to systematically test the hypothesis and explore a possible and biologically plausible network structure that reduces the correlation among inhibitory inputs on excitatory neurons, here we consider a two-layer feed-forward network. The input layer is composed of both inhibitory and excitatory neurons, whereas the output layer is composed only of excitatory neurons. In this network, we can artificially modulate correlated spike firings among the inhibitory input neurons.

**Figure 3:**
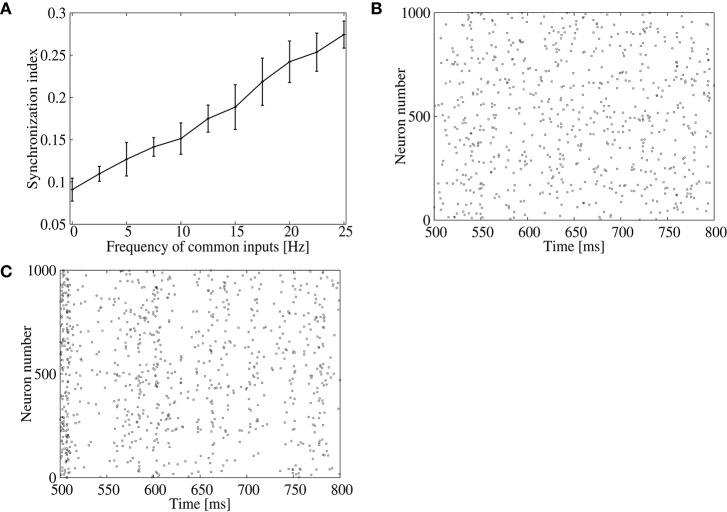
**Effect of inhibitory common inputs on synchronous firing of the feed-forward model**. **(A)** Dependence of the synchronization index between excitatory neurons on frequency of common inputs. The error bars indicate standard deviation of 5 realizations started from different random initial conditions. **(B,C)** Raster plots for excitatory neurons in the output layer. No common inputs were injected in **(B)**, whereas common inputs with a frequency of 25 Hz were injected in **(C)**.

We first confirm that the result of the previous subsection can be reproduced by increasing the correlated spike firings of inhibitory neurons in the input layer, which may induce synchronous firings of neurons in the output layer. As the frequency of the common inhibitory inputs is increased, the synchronization index of the output neurons increases almost linearly (Figure 3A). Indeed, the output spikes are less synchronized when the frequency of the common inhibitory input spikes is low (Figure 3B), while they show clear synchronization when the frequency is 25 Hz (Figure 3C).

Next, we explore a possible network mechanism that reduces the correlation of inhibitory inputs on output neurons to suppress their strong synchronization. Here, we focus on distribution of inhibitory connections on excitatory neurons. So far, we have assumed that strengths of inhibitory connections to output excitatory neurons are all the same, which may lead to a significant correlation among inhibitory inputs to them. Physiological experiments, however, reported that, in addition to heterogeneous EPSPs, inhibitory postsynaptic potentials (IPSPs) are also highly heterogeneous in the cortex (Miles and Wong, 1984; Holmgren et al., 2003; Chapeton et al., 2012). This heterogeneity can be a promising candidate to reduce the correlated inhibitory inputs on neurons and suppress the pathological synchronization in the cortex.

**Figure 4:**
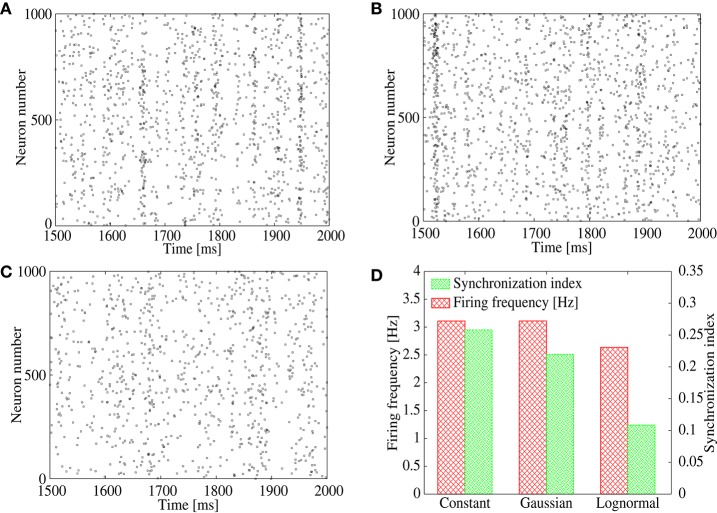
**(A–C)** Raster plot representing spikes of excitatory neurons in output layer of the feed-forward model. Their IPSPs are set to all the same in **(A)**, normally distributed in **(B)**, and lognormally distributed in **(C)**. **(D)** Firing frequency (red) and synchronization index (green) of the excitatory neurons corresponding to **(A–C)**.

To study the above possibility, we measure the synchronization index among neurons in the output layer for three types of feed-forward networks with different amplitude distribution of the IPSPs, (1) constant (i.e., the same value is shared by all inhibitory connections), (2) the *Gaussian* distribution, and (3) the lognormal distribution. For the network of (1), we use *G*_*I, j*∈*E*_ = 0.002, whereas, for the networks of (2) and (3), parameters of the synaptic distributions *G*_*I, j*∈*E*_ are adjusted so that the mean IPSP amplitude keeps the same value as that of (1). We set frequency of the common inhibitory spike inputs to a high value, 25 Hz, to mimic a possible correlated inhibitory spikes induced by the bidirectional correlation. Figure 4D shows the synchronization index and population firing rate of the neurons in the output layer. While the population firing rates are almost the same among the three networks, the synchronization index is low only for the network with the lognormal IPSP distribution. Indeed, the raster plots show that synchronized firing still remains in the networks with constant (Figure 4A) and *Gaussian* (Figure 4B) IPSP distributions, whereas firing pattern of the lognormal IPSP network is asynchronous (Figure 4C).

**Figure 5:**
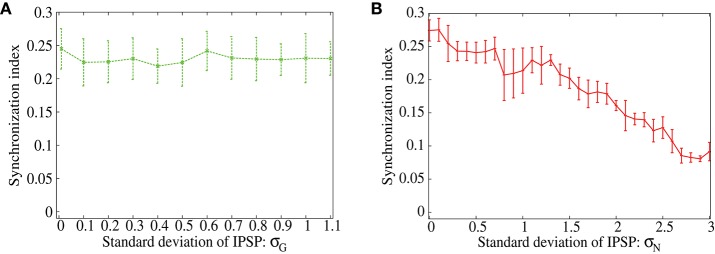
**(A,B)** Dependence of the synchronization index between excitatory neurons on standard deviation of the IPSPs. The error bars indicate standard deviation of 5 realizations started from different random initial conditions. Frequency of the common inputs is set to 25 Hz. The IPSPs are normally distributed in **(A)**, while they are lognormally distributed in **(B)**.

In order to show distinctive advantage of the lognormal IPSP distribution over the *Gaussian* distribution to suppress the correlation-induced synchronization, now we measure synchronization index of the output neurons for ranges of parameters that control width of the distributions (Figures 5A,B). We vary the standard deviation, σ_*G*_, of the *Gaussian* distribution and σ_*N*_ of the lognormal distribution (see Equation 2) with keeping the mean IPSP amplitudes the same. As shown in Figures 5A,B, the synchronization index decreases only at the first range of σ_*G*_ and stays at almost the same value even for sufficiently large values of σ_*G*_ in the case of *Gaussian* distribution. On the contrary, the synchronization index continues to decrease in the full range of σ_*N*_ for the lognormal distribution, and reaches a value < 0.1, that is remarkably small, in the network with the lognormal IPSP distribution. These results imply that modest heterogeneity of IPSP amplitudes, i.e., the *Gaussian* distribution, is not sufficient to suppress the synchronization, while the highly heterogeneous IPSP distribution, such as the lognormal distribution, works well.

### 3.3. Suppression of pathological synchronization in the recurrent network model of cortex

**Figure 6:**
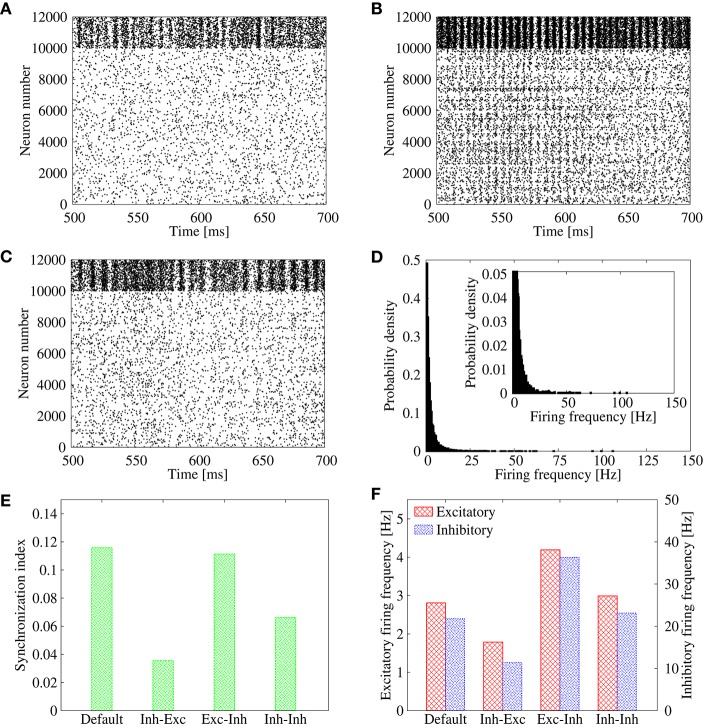
**(A–C)** Raster plots representing the firing pattern of the cortical network model in case of *R* = 0.35. For excitatory neurons, their spikes are indicated from neuron number 0 to 9999, whereas those of inhibitory neurons are indicated from neuron number 10, 000 to 11, 999. In addition to the lognormally distributed excitatory-to-excitatory connections, lognormal distribution is introduced also to inhibitory-to-excitatory connections in **(A)**, excitatory-to-inhibitory connections in **(B)**, and inhibitory-to-inhibitory connections in **(C)**. **(D)** Distribution of the firing frequencies of excitatory neurons corresponding to **(C)**. The inset represents the enlarged graph. **(E)** Synchronization indices of the excitatory neurons corresponding to Figure 1A (“Default”), Panel **(A)** (“Inh-Exc”), Panel **(B)** (“Exc-Inh”), and Panel **(C)** (“Inh-Inh”). **(F)** Mean firing rates of the excitatory (red) and inhibitory (blue) neurons corresponding to Figure 1A (“Default”), Panel **(A)** (“Inh-Exc”), Panel **(B)** (“Exc-Inh”), and Panel **(C)** (“Inh-Inh”).

In the previous subsection, we showed that the lognormal IPSP distribution efficiently suppresses the synchronization in the feed-forward network. Here, we apply the same strategy to the recurrent network, where correlation between bi-directional EPSPs results in pathological synchronization (*R* = 0.35; Figure 1A). In order to suppress the pathological synchronization, we introduce an additional lognormal distribution to the inhibitory-to-excitatory connections of the recurrent network (Figure 6A). In comparison, we also introduce a lognormal distribution to the excitatory-to-inhibitory connections (Figure 6B) or to the inhibitory-to-inhibitory connections (Figure 6C). Parameters of the lognormal distribution (i.e., μ, σ_*N*_) are adjusted so that the network maintains self-sustained activities and the neural firings are desynchronized. As shown in the raster plot, the synchronization is clearly suppressed in the case that the heterogeneity is introduced to the inhibitory-to-excitatory connections (Figure 6A). On the contrary, synchronization still remains in the case that the heterogeneity is introduced to the excitatory-to-inhibitory connections (Figure 6B). In the case that the heterogeneity is introduced to the inhibitory-to-inhibitory connections, the synchronization is also suppressed (Figure 6C). However, average firing rate of some neurons in the network is often extremely high, more than about 100 Hz (Figure 6D). In cortical networks, average firing rates of neurons in the resting state should be normally < 20 Hz. Note that if we readjust parameters of the lognormal IPSP distributions to avoid such extremely high firing rate, it destabilizes the spontaneous firings and results in disappearance of the spontaneous firing state. Figures 6E,F summarize these results.

**Figure 7:**
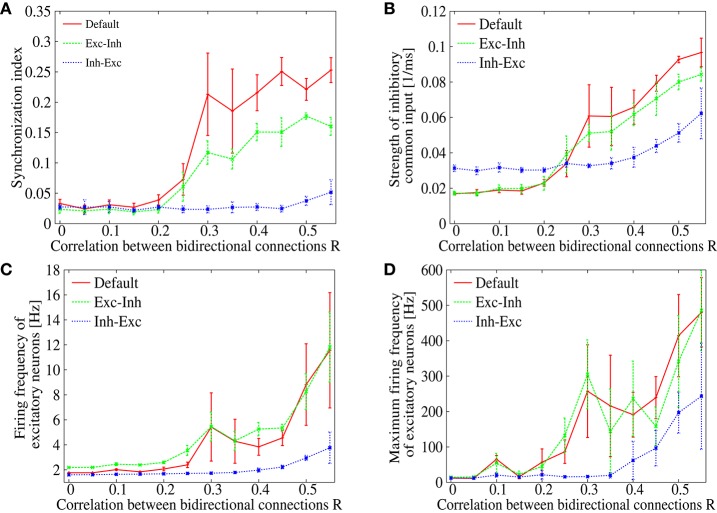
**(A)** Dependence of the synchronization index on correlation parameter *R*. The red solid line is identical to Figure 1A (“Default”), whereas blue or green dotted line corresponds to the case that lognormal distribution is introduced to inhibitory-to-excitatory connections (“Inh-Exc”) or excitatory-to-inhibitory (“Exc-Inh”) connections. The error bars indicate standard deviation of 5 realizations started from different random initial conditions. **(B)** Dependence of the strength of the common inhibitory input *CI* on the correlation parameter *R*. **(C)** Dependence of the mean firing frequencies of the excitatory neurons on the correlation parameter *R*. **(D)** Dependence of the maximum firing frequencies of the excitatory neurons on the correlation parameter *R*.

In order to see the robustness of suppressing the pathological synchronization by the additional lognormal distribution of the inhibitory-to-excitatory connection strengths, we measure the synchronization index of the spontaneous activity in recurrent networks with various correlation parameter *R*. Figure 7A compares three cases: (i) in addition to the lognormal distribution in the excitatory-to-excitatory connections, no lognormal distribution is introduced (red), (ii) lognormal distribution is introduced to the inhibitory-to-excitatory connections (blue), and (iii) lognormal distribution is introduced to the excitatory-to-inhibitory connections (green). In case (i), the synchronization index starts to increase rapidly as the correlation *R* is increased from about 0.25 (as we have already seen in Figure 1A). Similarly, in case (iii), the synchronization index starts to increase around *R* > 0.25. In contrast, in case (ii), the synchronization index is clearly suppressed over almost whole range of the correlation *R*. The lognormal distribution introduced to the inhibitory-to-excitatory connections, therefore, has a distinctive effect on desynchronization in the network model with biologically plausible features of non-random synaptic connectivity. Although, several studies reported that heterogeneities decrease synchrony in cortical networks (White et al., 1998; Golomb and Hansel, 2000; Neltner et al., 2000), no emphasis has been made on the importance of a long-tailed distribution, e.g., the lognormal distribution, as the form of heterogeneity.

The mechanism of desynchronization is considered to be due to the heterogeneity in the inhibitory-to-excitatory connections, which disperse the commonality of the spike inputs and weaken the strength of the common inhibitory drive. Figure 7B shows dependence of the common input strength *CI* on the correlation parameter *R*. In cases (i) and (iii), the common input strength increases monotonously as the correlation *R* is increased (red and green). The common input strength stayed in a low level up to *R* < 0.2 and then it increases rapidly as the correlation *R* is further increased. This coincides very well with the onset of synchronization, in which the level of synchrony increases in Figure 7A (red and green). In contrast, only a minor increase in the common input strength is observed in case (ii) (blue line of Figure 7B). Thus, the strength of the common input drive *CI* may provide a good index to measure the causality of the synchronized dynamics. As shown in Figures 7C,D, firing rates of the excitatory neurons are kept in a low level in case (ii) (blue). The firing rates, on the other hand, reach to an abnormal range in the other two cases (red and green). This implies that the heterogeneity in the inhibitory-to-excitatory connections is effective not only for suppressing the synchrony but also for maintaining the firing rates in a normal frequency range.

## 4. Discussion

In this paper, pathological synchronization of spontaneous firings and a possible mechanism to suppress them in a cortical network model with biologically plausible non-random features of connectivity has been investigated. While the networks with coexistence of many weak and a few extremely strong excitatory synapses, or the lognormal EPSP distribution, can sustain spontaneous asynchronous irregular firing, introduction of experimentally observed positive correlation between bidirectional excitatory connections destabilizes the asynchronous activity and induces pathological synchronization among spontaneous neural firings. We show that the additional lognormal distribution in inhibitory-to-excitatory connections efficiently suppresses the pathological synchronization. The heterogeneous IPSPs induce a large response variability among excitatory neurons even when they are driven by highly correlated inhibitory inputs. In the studies of sensory neurons, effects of correlated input to neurons have been widely studied. For instance, it has been reported that the correlated inputs induce synchronous firings among neurons (Mainen and Sejnowski, 1995; Galán et al., 2006; de La Rocha et al., 2007; Doiron et al., 2016). Our present study reveals that correlated input from inhibitory neurons, that is indirectly strengthened by the bidirectional excitatory correlations, is the major cause of the pathological synchronization in networks with the long-tailed and correlated connectivity of excitatory synapses. We confirm validity of the additional heterogeneity in inhibitory connections in both a feed-forward and a biologically plausible model of the cortical network. Our result provides a functional role of highly heterogeneous distribution of strengths in inhibitory connections (Miles and Wong, 1984; Chapeton et al., 2012) that avoids pathological activity and ensures stable asynchronous irregular state in the cortex.

It should be noted that the present study is based on numerical simulations, the results of which may depend upon the parameter setting of the neural network model. The present setting, however, has been configured cautiously in good correspondence with mathematical analysis in the previous study (Teramae et al., 2012) and the newly added correlation between bidirectional connections are based on the physiological measurement (Song et al., 2005). Moreover, tendency of the network dynamics that the inhibitory neurons are more strongly synchronized with each other than the excitatory neurons (Figure 1C) is consistent with the experimental observation (Hasenstaub et al., 2005). It is therefore reasonable to consider that synchronized inputs from the inhibitory neurons are the primary cause of inducing synchronous firings among the excitatory neurons. It might be plausible to conclude that introduction of the heterogeneity to the inhibitory connections, which weakens the strength of the common inhibitory drive, provides an efficient way of suppressing the synchronous firings of the excitatory neurons.

It could be a significant future subject to study relationships between bidirectional excitatory correlation, heterogeneity among IPSPs, and synchronized firings observed during developmental process of the brain. It has been reported that cortical neurons show synchronized spontaneous activity both *in vivo* (Golshani et al., 2009) and *in vitro* (Corlew et al., 2004) in neonatal stage. Interestingly, the key differences in network structure between premature and mature brains are closely related to our results. First, connection probability between neurons in premature brain is generally higher than that in mature brain (Chechik et al., 1999; Paolicelli et al., 2011). The dense connectivity may imply highly correlated bidirectional couplings between excitatory neurons, where the connectivity gets sparse in developmental process probably due to synaptic plasticity. Second, GABAergic interneurons give excitatory actions to postsynaptic neurons rather than inhibitory actions in immature brain (Ben-Ari, 2002; Owens and Kriegstein, 2002). The excitatory actions may increase neural firing frequencies as observed in the pathological firings of our model. Reversal potential of GABA, which is initially in the level of depolarized membrane potential, shifts to mature hyperpolarized level in developmental process. Thus, the network structure such as correlated connections and heterogeneous inputs may largely change during development. How developmental process and synaptic plasticity balance between correlation of bidirectional connectivity and inhibitory heterogeneity to compatibly realize spontaneous “synchronous” firing of immature brain as well as spontaneous “asynchronous” irregular firing of mature brain is one of the most important future subjects.

Another subject that may relate to the present study is epileptic seizure. Decrease in GABAergic inhibition is known to cause epilepsy in experiment, for instance, in temporal lobe (Cossart et al., 2001; Wendling et al., 2002). Possible underlying mechanism of the epilepsy can be an imbalance between excitatory and inhibitory connections, which are well balanced in normal cortex (McCormick and Contreras, 2001). In addition to the collapse of the net balance, our results also suggest that decreased heterogeneity in inhibitory connections can be an alternative cause of the pathological synchronization. Even when the net strength of inhibitory inputs are balanced with that of excitatory ones, if the heterogeneity is broken by some reason, it can strengthen a potential correlation among the inhibitory inputs to neurons and bring the whole network to a strong synchronization with high firing rate. Such abnormal state should be quite similar to epileptic seizure. While the relationship between pathological synchronization of the model and actual epileptic seizure in cortex remains unclear, it must be worth while to study possible roles of heterogeneity of inhibitory connection strengths to prevent epilepsy in the brain.

## Author contributions

JT and IT designed the study and discussed the results. HK performed the model simulations and analyzed the data. JT, IT, and HK wrote the text.

## Funding

This work was partially supported by the Ministry of Internal Affairs and Communications with a contract entitled “R&D for fundamental technology for energy-saving network control compatible to changing communication status” in FY2014, as well as by KAKENHI (No. 25430028, No. 26286086, No. 25293053, No. 16K00343, No. 16H01719).

### Conflict of interest statement

The authors declare that the research was conducted in the absence of any commercial or financial relationships that could be construed as a potential conflict of interest.
